# A 3D Microfluidic Chip for Electrochemical Detection of Hydrolysed Nucleic Bases by a Modified Glassy Carbon Electrode

**DOI:** 10.3390/s150202438

**Published:** 2015-01-22

**Authors:** Jana Vlachova, Katerina Tmejova, Pavel Kopel, Maria Korabik, Jan Zitka, David Hynek, Jindrich Kynicky, Vojtech Adam, Rene Kizek

**Affiliations:** 1 Department of Chemistry and Biochemistry, Faculty of Agronomy, Mendel University in Brno, Zemedelska 1, Brno CZ-613 00, Czech Republic; E-Mails: jaja.vlachova@gmail.com (J.V.); severova@centrum.cz (K.T.); paulko@centrum.cz (P.K.); zitka12@gmail.com (J.Z.); d.hynek@email.cz (D.H.); vojtech.adam@mendelu.cz (V.A.); 2 Central European Institute of Technology, Brno University of Technology, Technicka 3058/10, Brno CZ-616 00, Czech Republic; 3 Faculty of Chemistry, University of Wroclaw, 14 Joliot-Curie, Wroclaw PL-50383, Poland; E-Mail: maria.korabik@chem.uni.wroc.pl; 4 Karel Englis College, Sujanovo nam. 356/1, Brno CZ-602 00, Czech Republic; E-Mail: jindrak@email.cz

**Keywords:** biosensor, lab-on-chip, electrochemistry, graphene, modification, hydrolysis, trithiocyanuric acid

## Abstract

Modification of carbon materials, especially graphene-based materials, has wide applications in electrochemical detection such as electrochemical lab-on-chip devices. A glassy carbon electrode (GCE) modified with chemically alternated graphene oxide was used as a working electrode (glassy carbon modified by graphene oxide with sulphur containing compounds and Nafion) for detection of nucleobases in hydrolysed samples (HCl pH = 2.9, 100 °C, 1 h, neutralization by NaOH). It was found out that modification, especially with trithiocyanuric acid, increased the sensitivity of detection in comparison with pure GCE. All processes were finally implemented in a microfluidic chip formed with a 3D printer by fused deposition modelling technology. As a material for chip fabrication, acrylonitrile butadiene styrene was chosen because of its mechanical and chemical stability. The chip contained the one chamber for the hydrolysis of the nucleic acid and another for the electrochemical detection by the modified GCE. This chamber was fabricated to allow for replacement of the GCE.

## 1. Introduction

The microfluidic systems called lab-on-chip devices enables one to create fast and low cost detection systems with ultralow sample consumption [1]. Due to the fact that the electrochemical detection is still very attractive for its high sensitivity and inherent simplicity [2–4], some of these are often used to construct the mentioned microfluidic systems, including those aimed at detection of viruses [5] or DNA/RNA nucleobases [6,7]. However, the sensitivity of such microfluidic devices is still an issue. In this field, the utilization of various electrode surfaces such as carbon-based modifications and boron-doped diamond electrodes have enhanced the sensitivity and reproducibility [8]. For electrochemical detection of nucleobases by a glassy carbon electrode (GCE), the modification of graphene-based compounds altered by Nafion [9] or Nafion with TiO_2_ [10] has been also used to increase the sensitivity.

Graphene oxide (GO) is a multilayer system created by the oxidation of graphite and therefore oxygenated functionalities are introduced in the graphite structure [11]. This fact causes hydrophilicity and GO becomes very well soluble in water or other solvents utilized for numerous purposes [12–14]. GO has been widely applied for modification [15,16] of glassy carbon electrodes [17] used for the detection of metal ions, proteins, DNA mutations and other compounds [18].

Trithiocyanuric acid (ttc), also referred to as trimercaptotriazine, has a symmetric structure with three nitrogen atoms in a ring and three sulphur atoms connected to carbons, whereby these six atoms are highly reactive and can form bridges connecting central atoms. To illustrate the importance of trithiocyanuric acid, its use as a remediation agent for removal of heavy metals from industrial waters should be mentioned [19–21]. Removal of residual palladium from reaction mixtures in drug production is an especially important application of ttcNa_3_ [22–24]. This acid was also applied in plating processes, in production of composite materials with metals and rubbers and as an anticorrosion agent [25–28].

In this work, modified graphene oxide was used to develop a simple and sensitive method for detection of adenine in a hydrolysed miRNA sample. The modified graphene oxide was firstly dissolved in dimethylformamide and subsequently modified by Nafion and sonicated in an ultrasonic bath. The mixture was deposited on a glassy carbon surface. This way the layer for each measurement was prepared. The described modification allows adenine detection in the hydrolysed miRNA sample and it was possible to detect other nucleic bases too. Moreover, hydrolysis and electrochemical detection was carried out within a microfluidic chip with a chamber that allows repeatable input and output of the modified working electrode.

## 2. Experimental Section

### 2.1. Chemicals

**Figure 1.**
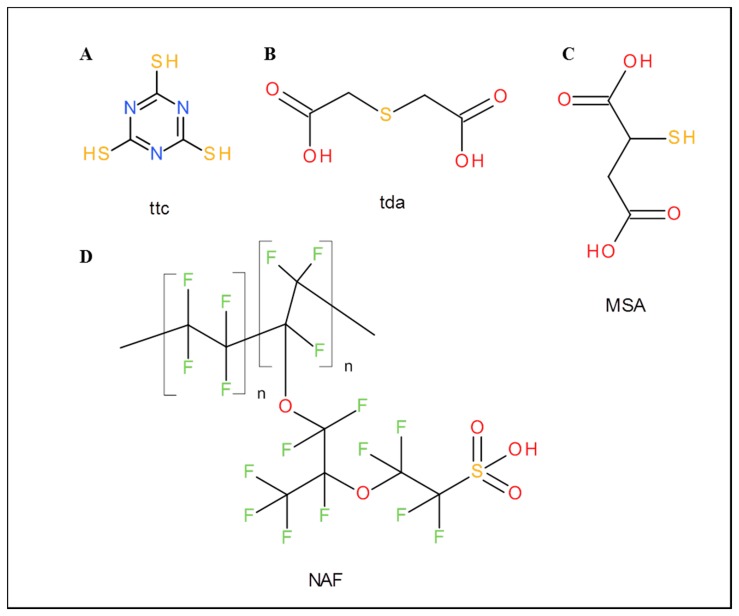
Schemes of compounds used for modification of graphene oxide. (**A**) ttc; (**B**) tda; (**C**) MSA; (**D**) Nafion (NAF).

Ttc, thiodiacetic acid (tda), mercaptosuccinic acid (MSA), Nafion 117 (formulas are shown in Figure 1), titanium(IV) isopropoxide, graphite flakes, NaNO_3_, KMnO_4_, sodium acetate trihydrate, acetic acid, Hg(NO_3_)_2_, water and other chemicals were purchased from Sigma-Aldrich (St. Louis, MO, USA) in ACS purity (chemicals meet the specifications of the American Chemical Society). MiRNA was also purchased from Sigma-Aldrich and its sequence was 5′ UAA GGC ACG CGG UGA AUG CCA AAA AAA AAA 3′. Standard nucleobases were adenine and cytosine (MP Biomedicals, Santa Ana, CA, USA), guanine and uracil (Sigma-Aldrich, St. Louis, MO, USA).

### 2.2. GO Modifications

#### 2.2.1. Preparation of GO

GO was prepared from graphite flakes by the Hummers method [29]. Briefly, graphite (2 g), NaNO_3_ (1 g) and KMnO_4_ (6 g) were added with stirring to concentrated H_2_SO_4_ (96%, *w*/*w*, 46 mL) placed in an ice bath. The mixture was stirred overnight with gradual temperature growth. Water (400 mL) was slowly added and the mixture was heated at 90 °C for 2 h. Subsequently, H_2_O_2_ (3%, *v*/*v*) was slowly added till colour turned to yellow. The product was decanted and washed several times with water. The final volume of the GO suspension was 200 mL.

#### 2.2.2. Preparation of Modified GO (GO-ttc, GO-tda, and GO-MSA)

In general, the samples were prepared as described in the previous paragraph by the following method: GO suspension (25 mL) in a 100 mL beaker was mixed with thiol compound (ttc, tda, or MSA in the amount of 0.5 g) dissolved in water (5 mL). Samples were diluted with water and solution was shaken on orbital shaker overnight. The next step was decantation and washing several times with water. Samples were collected by centrifugation and dried at 60 °C.

#### 2.2.3. Preparation of GO-TiO_2_ Solution

GO-ttc (1 mg) in water: ethanol (1:1) mixture (0.5 mL) was sonicated (1 h). Titanium (IV) isopropoxide (40 μL) was added and sonicated for 2 h. The sample was centrifuged and solvent was discarded. Dimethylformamide was added (0.85 mL) and sonicated for 1 h. Finally, Nafion 117 (100 μL) was added and sample was sonicated for 0.5 h. This solution was used for glassy carbon modification.

#### 2.2.4. Preparation of GO-thiol-Nafion Solutions (GO-ttc-NAF, GO-tda-NAF, and GO-MSA-NAF)

These solutions were prepared as follows. 1 mg of GO-thiol was suspended and sonicated in dimethylformamide (1 mL) for 2 h. Then, Nafion 117 (100 μL) was added and sonicated for 0.5 h. This solution was used for glassy carbon modification.

### 2.3. X-ray Fluorescence Analysis (XRF)

The samples were analysed on Spectro Xepos (Spectro Analytical Instruments, Kleve, Germany) using anode X-ray tube with Pd anode working at a voltage of 44.69 kV and a current of 0.55 mA. Signals were detected with Barkla scatter aluminium oxide for 300 s. For excitation three secondary targets (Mo, Al_2_O_3_ and high-ordered pyrolithic graphite crystal) were used. The excitation geometry was 90°. The samples were measured through the polyethylene bottle side wall 20 mm above the bottom. The Spectro Xepos software and TurboQuant method were applied for data analysis.

### 2.4. Elemental Detection

C, H, N and S detection of samples was carried out on Flash 2000 (Thermo Scientific, Waltham, MA, USA).

### 2.5. Fourier Transformation Infrared Spectroscopy (FTIR)

FTIR study was performed over the range of wavenumber 4000–400 cm^−1^ by a Bruker Vertex 70 FTIR spectrometer (Bruker, Ettlingen, Germany) using KBr pellets.

### 2.6. Sample Preparation for Electrochemical Detection of Nucleic Bases

24 μL of HCl (pH = 2.9) was added to 24 μL of 22 μM miRNA and mixture was incubated at 99 °C for 1 h in Thermomixer comfort (Eppendorf Czech & Slovakia s.r.o., Ricany, Czech Republic). After hydrolysis the mixture was neutralized by equimolar quantity of NaOH. Hydrolysed samples were analysed electrochemically.

### 2.7. Electrochemical Detection

Electrochemical detection was carried out using a CH Instruments Electrochemical Workstation (CH Instruments, Bee Cave, TX, USA) and glass cell with three electrodes. Ag/AgCl/3M KCl was used as a reference electrode and platinum as a counter electrode. As a working electrode, modified GCE was used. This GCE was modified by various types of chemically modified graphene oxide (Table 1, Figure 1). Five μL of chemically modified graphene oxide in dimethylformamide was applied on the GCE and dried with a hair drier for 15 min. For all measurement differential pulse voltammetry was used and measurement parameters were as follows: initial potential 0.2 V, final potential 1.5 V for adenine and 1.8 V for simultaneous detection of bases and hydrolysed sample, step potential 0.004, modulation amplitude 0.05 V, pulse width 0.2 s, sampling width 0.0167 s, pulse period 0.5 s, quiet time 10 s. All experiments were performed at room temperature. As the supporting electrolyte the acetate buffer (pH = 5, 0.2 M CH_3_COOH and 0.2 M CH_3_COONa) was used. 1 ml of electrolyte was used for adenine detection and 0.5 mL for simultaneous detection of bases and hydrolysed sample. The limit of detection was calculated by $\text{LOD}=\frac{3.3\times SD}{S}$, *SD* = standard deviation of the response and *S* = slope of the calibration curve.

### 2.8. Microfluidic Detection System

Electrodes were placed to a holder fabricated by using a 3D printer (Profi3Dmaker, 3D Factories, Straznice, Czech Republic) using Fused Deposition Modelling (FDM) technology. For electrochemical detection in the microfluidic system different electrodes were chosen due to their size. Standard Ag/AgCl/3M KCl was replaced with quasi-reference electrode as graphite lead and as a counter electrode a platinum electrode with diameter 0.5 mm (CH Instruments, Bee Cave, TX, USA) was used.

## 3. Results and Discussion

### 3.1. Characterisation of Graphene Modified Materials

#### 3.1.1. X-Ray Fluorescence Spectroscopy and Elemental Analysis

**Figure 2.**
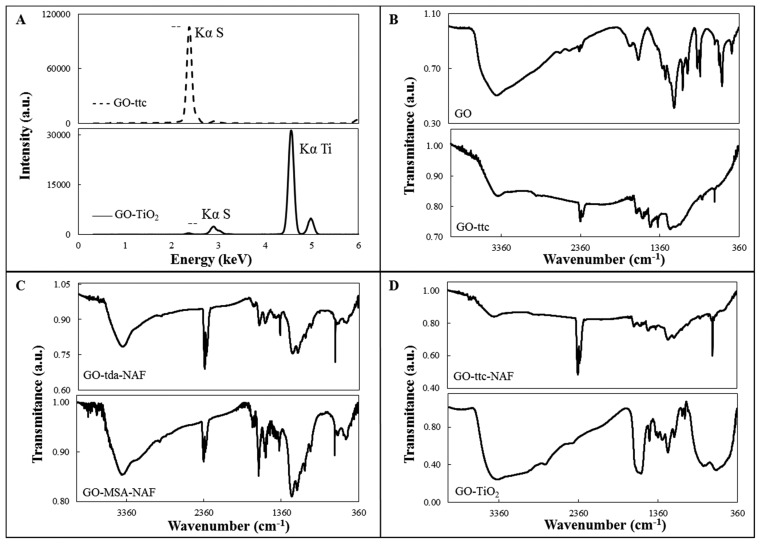
Characterization of graphene oxide modified materials. (**A**) XRF analysis of GO-ttc (dashed line) and GO-TiO_2_ (solid line); (**B**–**D**) IR spectra of studied materials.

Modified GO was prepared by mixing of a water suspension of GO with sulphur-containing compounds. It was easily seen that the samples changed in colour from light brown to deep brown or black during overnight mixing. Prepared samples were decanted and washed several times with water to remove excess reagents. Solid samples were obtained by centrifugation and drying. Composition of the modified GO was proved by X-ray fluorescence spectroscopy and elemental analysis. The presence of sulphur in all the samples was proved by peak characteristic for S_Kα_, which appeared in all XRF spectra. The peak position was always the same, whereas the intensity of the peak was changing. That is why only one typical spectrum is shown in Figure 2A (top) for GO-ttc. XRF spectrum of GO-TiO_2_, where peak Ti_Kα_ was also observed (Figure 2A (bottom)) and thus presence of Ti in the sample was unambiguously proved, was the only exception. C, H, N, and S elemental analysis of samples also proved the presence of sulphur and content of carbon and nitrogen in the case of trithiocyanuric acid (Table 2).

#### 3.1.2. FTIR Analysis

FTIR spectra of GO and modified GO are shown in Figures 2B–D. The presence of oxygen containing groups such as carbonyl, carboxylic acid, epoxy and hydroxyl in the GO is proved by the presence of the corresponding characteristic peaks. These peaks can be attributed to ν(C=O) stretching (1663 cm^−1^), C-O-C stretching (1193 cm^−1^), ν(C-O) stretching (1072 cm^−1^) and a broad shoulder with a maximum at 3480 cm^−1^ can be assigned to vibration of hydroxyl groups [30]. The spectrum of GO modified with Nafion (GO-NAF) (not shown) was also measured. In the GO-NAF spectrum there is very broad band from 400–800 cm^−1^, probably due to CO vibrations, and very weak peaks in the 1212–1232 cm^−1^, 1150–1160 cm^−1^ and 1056–1105 cm^−1^ regions that can be assigned to ν_as_(CF_2_), ν_s_(CF_2_) and ν_s_(SO), respectively [31]. The same peaks, of weak or medium intensity, were found in the spectra of Nafion-containing samples. In the spectra of all compounds, except for the GO spectrum, there is very strong peak near 670 cm^−1^ characteristic for sulphur-containing compounds, attributable to ν(C-S). In the spectra of GO-tda-NAF and GO-MSA-NAF (Figure 2C) there are, in comparison with the GO spectrum, more intense peaks near 1560 and 1388 cm^−1^, which can be attributed to ν_as_(COO) and ν_s_(COO) vibrations, respectively [32]. In the spectra of ttc-modified GO, peaks in the 1248–1265 and 1529–1589 cm^−1^ regions were found, which can be attributed to ν(C-N) vibrations of the heterocyclic ring and peaks near 870 cm^−1^ which are most probably connected with ν(C-S) vibrations [33]. In the spectrum of GO-TiO_2_ a shoulder from 400 to 670 cm^−1^ was observed, which is probably caused by the presence of the Ti-O stretching vibration. Thus, the modification of GO by TiO_2_ on the surface was confirmed.

### 3.2. Electrochemical Detection of Nucleobases in Electrochemical Cell

**Figure 3.**
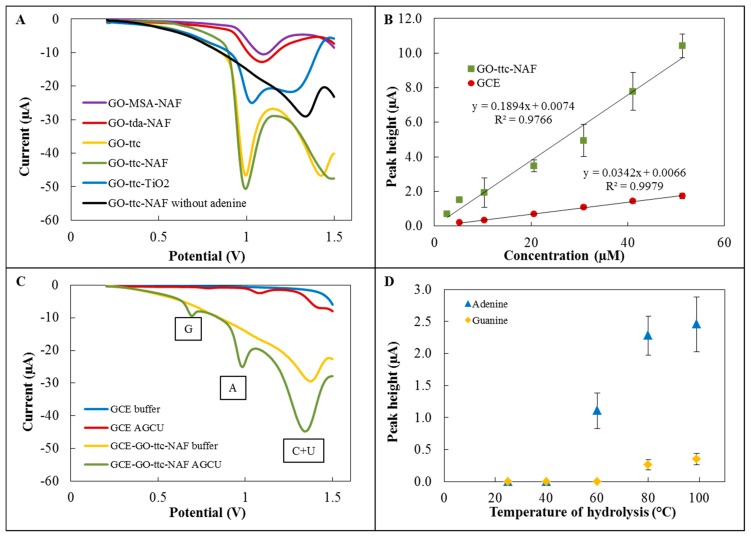
(**A**) Detection of 200 μM adenine on GCE modified by different graphene oxide; (**B**) Calibration curve of adenine on unmodified GCE and GCE modified by GO-ttc-NAF; repeated in triplicates; (**C**) Simultaneous detection of guanine (262 μM), adenine (51 μM) and cytosine (273 μM) with uracil (255 μM) by unmodified GCE and GCE modified by GO-ttc-NAF; (**D**) Optimization of hydrolysis temperature measured by GCE modified with GO-ttc-NAF, concentration of adenine and guanine was 9 μM and 4 μM respectively.

The first step of the detection part of the experiment involved testing of five variously modified graphene oxides for adenine detection. The electrochemical detection was performed by differential pulse voltammetry using a GCE modified with specific graphene oxides. Figure 3A depicts voltammograms of these modifications with the characteristic peak for adenine, which was detected at approximately 1.0 V. The highest sensitivities were found for modifications with trithiocyanuric acid. According to [9,10], we attempted to improve the sensitivity by Nafion and TiO_2_, respectively. In the case of modification by TiO_2_, the sensitivity decreased, but for GO-ttc-NAF higher sensitivity than without Nafion was found. Voltammograms of GO-ttc, GO-ttc-NAF and GO-TiO_2_ show another peak at a potential of about 1.4 V. This peak is probably connected with oxidation of the material itself. The high sensitivity of trithiocyanuric acid to nucleobases was probably caused by its ability to form hydrogen bonds with compounds containing nitrogen [34]. The addition of Nafion had a few supplemental positive results. It prevented graphene from aggregation and increased the stability of the immobilisation of graphene on GCE. The negative charge of Nafion can facilitate adsorption of positively charged nucleobases. Nafion also increased the oxidation signal due to the easier electron transfer [9]. Based on these advantages we assume this modification GO-ttc-NAF has great potential for wide electrochemical application. The next step was the determination of calibration curves for adenine on unmodified GCE and GCE modified by GO-ttc-NAF. Both curves fitted by linear regression are depicted in Figure 3B. Analytical parameters like regression parameters, standard deviation, limit of detection and quantification are listed in Tables 3 and 4. We observed quite similar limits of detection for unmodified and modified GCE. Although the sensitivity of applied electrochemical method using modified GCE was five times higher, the dissimilar surface of the modified electrode caused a high standard deviation which resulted in a detection limit decrease.

Furthermore, simultaneous detection of all nucleobases was performed on GCE modified by GO-ttc-NAF. Figure 3C shows a comparison between detection on this modified GCE and unmodified GCE. By using of GCE modified by GO-ttc-NAF three peaks may be observed. The peaks at potentials of 0.75 V and 1.0 V belong to guanine and adenine, respectively. The last peak at a potential of about 1.4 V was a signal of cytosine and uracil, which had peaks at the same potential and thus it is not possible to distinguish between them. The one disadvantage of GCE modified by GO-ttc-NAF was the peak of the material itself, which occurred at the same potential as the peak for cytosine and uracil. For this reason it was not possible to determine small amounts of cytosine and uracil. It should be noted that the best sensitivity was determined for adenine which shows a very high peak at a concentration of 51 μM in contrast to other nucleobases, whose concentrations were about 260 μM.

### 3.3. Optimization of the Hydrolysis Process

The optimization was carried out for adenine and guanine detection at different temperatures of hydrolysis as follows: 25 °C, 40 °C, 60 °C, 80 °C and 99 °C (Figure 3D). At 25 °C and 40 °C no signal was observed for either adenine or guanine. At 60 °C the adenine peak occurred, but the peak for guanine was not observed. Both peaks were found for temperatures of hydrolysis of 80 °C and 99 °C. The difference between the signals was insignificant (about 0.1 μA).

### 3.4. Microfluidic System

**Figure 4.**
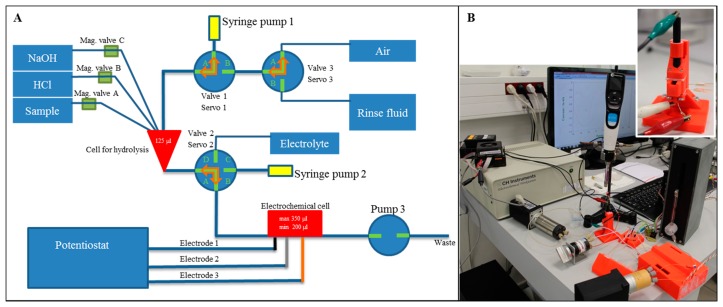
(**A**) The scheme of the microfluidic system composed of the hydrolysis and detection parts; (**B**) Picture of the fabricated microfluidic system and detailed picture of the chip with integrated electrodes.

Connection of the hydrolysis process and electrochemical detection of nucleobases was the great challenge and therefore microfluidic system was designed and fabricated. The microfluidic system was composed of two basic parts: the first part was for hydrolysis, the second one for the electrochemical detection. The principle scheme of the suggested system is shown in Figure 4A. The fluidic system was realized by plastic tubes. The liquid movements were done using three syringe pumps, three three-step valves and three magnetic valves. The microfluidic chip was printed by a 3D printer and as a material for printing, acrylonitrile butadiene styrene was chosen because of its thermal and chemical stability.

#### 3.4.1. Hydrolysis Part

The first part of the system designed for the hydrolysis process contained three inputs and one output to the second part of the system. The chamber comprised three basic parts as follows: model cover, body and heaters case. The hydrolysis chamber had a volume of 125 μL and it was cone-shaped to ensure the greatest possible outflow. In the body, there were PT100 temperature sensors (which resistance is linearly dependent on temperature changes). These three sensors were placed at different radius from the centre, where the sample was placed. The body had a cylindrical shape with a height of 8 mm and 30 mm in diameter. In the body there were three screw holes with M4 thread.

The cover had a drum shape with 30 mm in diameter and 6 mm in height. There were holes for input to the hydrolysis chamber. It also contained a recess for three M4 Allen screws, which connected the cover and the body tightly together to protect samples from evaporation. The major advantage was printing of seals from flexible material which prevented the evaporation.

The heater case had a square shape with side length of 50 mm and height of 15 mm. In the centre of heater case there was a space for the body and the cover. This space was in the form of rolls with 31 mm in diameter and it is 14 mm high. Due to the larger size, it was possible to glue an adhesive CU tape on the body to spread a heat uniformly to the centre of body. The cabling for four heaters led from the centre of the heater case. Heaters were at the same distance from the centre axis and from each other. They were placed 20 mm from the centre and at the same height as the centre cell in the body. Cabling was led to one side of the heaters case and then on the other side, next was led out from the body. The part of the system designed to hydrolyse the sample was controlled by a unit which regulated the temperature in the chip at the required temperature (100 °C) by 2 °C hysteresis. The control unit regulated the temperature on the outer part of chamber (12 mm from the centre), in the central part of body (8 mm from the centre) and also directly near to the hydrolysis chamber. Hence it was possible easily regulate the thermal transmittance and short-term overheating did not exceeded over than 1.5 °C. Heaters were turned on by a 12 V bipolar transistor.

#### 3.4.2. Detection Part

The second part of the chip was designed for electrochemical detection. There were two inputs and one output. The main parts of the detection unit were made by a 3D printer from acrylonitrile butadiene styrene material as follows: holder, detection cell an pressure cover. The whole construct was completed by two sealing parts. The seals were also made by the 3D printer but from a flexible material (Elastic Printplus Natural from 3DFactories). The seals had a square shape and the height was 0.9 mm. The holes for electrode were in the centres of individual seals and these holes had a circular shape with 6 or 3 mm in diameter.

The model holder had a wedge shape and it had to hold the detection cell in the angle 10° from the horizontal pad on which it stood. It also contained holes for M3 screws which pressed all the parts tightly together. In the holder, there was the hole for the working electrode (modified glassy carbon) with 6.3 mm in diameter. This hole had larger diameter (6.5 mm) for easier replacement of electrode.

The model detection cell had rectangular ground plan with sides of dimension 15 × 20 mm and 7 mm height. It contained four holes for mounting M3 screws and a detection chamber with a volume of 430 μL. The cell surface was smoothed by a borer with 6 mm in diameter. The pressure cover had a square shape with size 15 mm, the height was 5 mm and there were holes for holding by M3 screws. Holes with 0.5 mm in diameter were drilled for electrodes.

The whole chip part for detection was assembled using four screws M3 and nuts. Thanks to these screws the tightness of the whole chip was ensured. The working electrode was led from below the chip and it was placed in a position where the electrode was always immersed in electrolyte (minimal volume 200 μL). Other two electrodes were placed opposite the main electrode and their position was near the bottom of the detection cell, so these electrodes were also always completely immersed in the electrolyte. The electrodes did not touch each other. Two inputs in the form of insulin needles led to the detection cell. One from this input served as an output (the input closer to the working electrode, because there was the lowest point of whole model). All electrodes were connected to the Electrochemical Workstation (Ch Instruments, Bee Cave, TX, USA). The basic system operation is described in detail in the Supplementary Material.

### 3.5. Analysis of Sample in Microfluidic System

**Figure 5.**
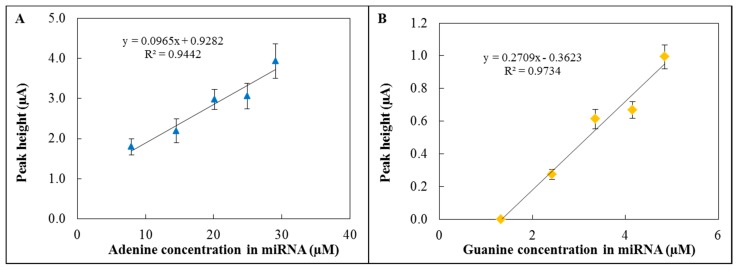
Calibration curves related to the electrochemical detection of (**A**) adenine and (**B**) guanine in hydrolysed miRNA in the microfluidic system; repeated in triplicate. Presented amounts of adenine and guanine are based on miRNA sequence recalculation.

The hydrolysis of miRNA and subsequent electrochemical detection were performed using the 3D fabricated microfluidic system (Figure 4B). The hydrolysis was carried out in hydrolysis cell by HCl (pH = 2.9) at 99 °C for 1 hour. Then the sample was neutralized by an equimolar amount of NaOH and the whole volume was transported to the electrochemical detection cell. A graphite electrode was used as a reference electrode, platinum as a counter electrode and GCE modified by GO-ttc-NAF as a working electrode. The oxidation peak of adenine occurred at 0.85 V (Figure 5A) and the peak of guanine (Figure 5B) occurred at 0.54 V; all measurements were done three times. The reason why we were also interested in guanine oxidation peak evaluation was the transformation of adenine to guanine nucleobases during the hydrolysis process. For the lowest concentration of miRNA the peak for adenine was detected, but this was not the case of guanine. Therefore the presented calibration curve for guanine (Figure 5B) contains the point for zero peak height. Both peaks were evaluated according to various concentrations of hydrolysed miRNA. The concentrations of adenine and guanine presented in Figure 5A and 5B were recalculated according to the amount of each nucleobase in miRNA (15 times adenine, seven times guanine). Both peaks increased with increasing concentration of hydrolysed miRNA which proved the functionality of the proposed microfluidic detection system. The obtained sensitivity of detection (Figure 5A) in the microfluidic system was lower in comparison to the determination in an electrochemical cell (Figure 3B) due to the smaller auxiliary electrode surface.

## 5. Conclusions

Due to the growing interest in miRNA and its detection, the focus on technologies which allow the detection of these compounds is understandable. The electrochemical determination technique is among the more infrequently used methods. In this article a new lab-on-chip device for electrochemical determination of nucleic bases in hydrolysed miRNA is presented. For detection, a modified graphene oxide electrode was used as working electrode resulting in an increased detection sensitivity compared to an unmodified graphene oxide electrode. This model provides user friendly possibility of nucleic acids bases detection, including steps that are done automatically in one system.

## Supplementary Materials



## Figures and Tables

**Table 1.** Chemical agents used for modification of graphene oxide.

**Abbreviation**	**Applied Modifiers**
GO-MSA-NAF	mercaptosuccinic acid and Nafion
GO-tda-NAF	thiodiacetic acid and Nafion
GO-ttc	trithiocyanuric acid
GO-ttc-NAF	trithiocyanuric acid and Nafion
GO-TiO_2_	trithiocyanuric acid, Nafion and titanium dioxide

**Table 2.** Elemental analysis results of various modified graphene oxides.

	**Nitrogen (%)**	**Carbon (%)**	**Hydrogen (%)**	**Sulphur (%)**
GO	0	84.15	0.44	0
GO-MSA	0	43.27	2.34	15.45
GO-tda	0	38.06	2.89	20.77
GO-ttc	10.14	52.95	1.35	25.95
GO-TiO_2_	6.56	34.12	0.78	16.02

**Table 3.** Analytical parameters of electrochemical detection of unmodified GCE, *n* = 3.

**Substance**	**Regression Equation**	**Linear Dynamic Range (μM)**	**Linear Dynamic Range (μg/mL)**	**R ^1^^,^^2^**	**LOD ^2^ (μM)**	**LOD (μg/mL)**	**LOQ ^3^ (μM)**	**LOQ (μg/mL)**	**RSD ^4^ (%)**
unmodified GCE	y = 0.0342x − 0.0066	5.18–51.30	0.70–6.93	0.9979	0.67	0.09	2.23	0.30	3.72

1 Ccoefficients of determination;

2 Limits of detection of detector (3 S/N);

3 Limits of quantification of detector (10 S/N);

4 Relative standard deviations.

**Table 4.** Analytical parameters of electrochemical detection of GCE modified by CO-ttc-NAF, *n* = 3.

**Substance**	**Regression Equation**	**Linear Dynamic Range (μM)**	**Linear Dynamic Range (μg/mL)**	**R ^1^^,^^2^**	**LOD ^2^ (μM)**	**LOD (μg/mL)**	**LOQ ^3^ (μM)**	**LOQ (μg/mL)**	**RSD ^4^ (%)**
modified GCE	y = 0.1894x − 0.0074	2.59–51.30	0.35–6.93	0.9766	0.66	0.09	2.21	0.30	5.95

1 Ccoefficients of determination;

2 Limits of detection of detector (3 S/N);

3 Limits of quantification of detector (10 S/N);

4 Relative standard deviations.
