# Supplementary material for: A 3D Microfluidic Chip for Electrochemical Detection of Hydrolysed Nucleic Bases by a Modified Glassy Carbon Electrode
Source: Sensors (Basel). 2015 Jan 22;15(2):2438–52. doi: 10.3390/s150202438 (PMC4367314; doi:10.3390/s150202438)
Supplement: Supplementary file 1 [file sensors-15-02438-s001.pdf]

OPEN ACCESS

sensors

ISSN 1424-8220

www.mdpi.com/journal/sensors

Supplementary Information

A 3D Microfluidic Chip for Electrochemical Detection of Hydrolysed Nucleic Bases by a Modified Glassy Carbon Electrode. *Sensors* 2015, *15*, 2438-2452

Jana Vlachova [1,2], Katerina Tmejova [1,2], Pavel Kopel [1,2], Maria Korabik [3], Jan Zitka [1], David Hynek [1,2], Jindrich Kynicky [4], Vojtech Adam [1,2] and Rene Kizek [1,2,*]

1 Department of Chemistry and Biochemistry, Faculty of Agronomy, Mendel University in Brno, Zemedelska 1, Brno CZ-613 00, Czech Republic; E-Mails: jaja.vlachova@gmail.com (J.V.); severova@centrum.cz (K.T.); paulko@centrum.cz (P.K.); zitka12@gmail.com (J.Z.); d.hynek@email.cz (D.H.); vojtech.adam@mendelu.cz (V.A.)

2 Central European Institute of Technology, Brno University of Technology, Technicka 3058/10, Brno CZ-616 00, Czech Republic

3 Faculty of Chemistry, University of Wroclaw, 14 Joliot-Curie, Wroclaw PL-50383, Poland; E-Mail: maria.korabik@chem.uni.wroc.pl

4 Karel Englis College, Sujanovo nam. 356/1, Brno CZ-602 00, Czech Republic; E-Mail: jindrak@email.cz

* Author to whom correspondence should be addressed; E-Mail: kizek@sci.muni.cz; Tel.: +420-5-4513-3350; Fax: +420-5-4521-2044.

Basic Systems Operations

The whole system was controlled by the unit with a program allowing elementary changes in the detection procedure. The main features were sample volume, HCl volume, NaOH volume, electrolyte volume and technical parameters such as hydrolysed period (TIME_H), temperature hydrolysis (TEMP) and area of tubes between hydrolysed and detection part (TRANSPORT). Before the detection process, tubes from hydrolysis solutions and electrolyte had to be immersed deep enough. The working unmodified electrode was inserted.

1. Magnetic valve A was opened and syringe pump1 sucked a small amount of sample to get the liquid to the end of tubing. Magnetic valve A was closed.
2. Magnetic valve B was opened and syringe pump1 sucked a small amount of sample to get the liquid to the end of tubing. Magnetic valve B was closed.
3. Magnetic valve C was opened and syringe pump1 sucked a small amount of sample to get the liquid to the end of tubing. Magnetic valve C was closed.

4. Servo 2 with valve 2 switched to position B and syringe pump 2 sucked a small volume of electrolyte to get the liquid to the end of tubing.
5. Servo 2 with valve 2 was switched to position C and syringe pump 2 pushed the liquid out.
6. Servo 2 with valve 2 was switched to position A.
7. Servo 3 with valve 3 was switched to position B, servo 1 with valve 1 was switched to position B and syringe pump 1 sucked rinse fluid to the maximal volume of the syringe pump.
8. Servo 1 with valve 1 was switched to position A and syringe pump 1 pushed rinse fluid out.
9. Servo 2 with valve 2 was switched to position C and syringe pump 1 sucked the volume back for washing of hydrolysis cell.
10. Syringe pump pushed liquid out back to zero volume of the syringe pump after 5 seconds .
11. Servo 3 with valve 3 was switched to position A, servo 1 with valve 1 was switched to position B and syringe pump 1 sucked in air to the maximal volume of the syringe pump.
12. Servo 1 with valve 1 was switched to position A and syringe pump 1 pushed rinse fluid out to the detection cell. Pump 3 sucked rinse fluid out to waste.

Detection Procedure

The initial conditions for the start of the detection process were: all pumps were in zero positions, all servos with valves were in position A, magnetic valves were closed, all solutions were sucked into the tubes and remaining tubes were washed and emptied. The main electrode was modified and inserted.

1. Magnetic valve A was opened and the syringe pump sucked in a defined volume of sample.
2. Magnetic valve A was closed, servo 1 with valve was switched to the position B and the syringe pump went to zero position.
3. Servo 1 with valve 1 was switched to position A, magnetic valve B was opened and pump 1 sucked in a defined amount of HCl.
4. Magnetic valve B was closed, servo 1 with valve 1 was switched to the position B and syringe pump 1 went to zero position.
5. Servo 1 with valve 1 was switched to position A, magnetic valve C was opened and syringe pump 1 sucked in a defined amount of NaOH.
6. Magnetic valve C was closed, servo1 with valve 1 was switched on to position B and syringe pump 1 went to zero position.
7. Control unit regulated temperature by sensors IT100 and heater sensors according to variable TEMP.
8. Twenty seconds before the end of hydrolysis servo 2 with valve 2 was switched to the position B and syringe pump 2 sucked in a defined volume of electrolyte.
9. Servo 2 with valve 2 was switched to the position C and syringe pump 2 pushed the liquid out.
10. Servo 2 with valve 2 was switched to the position A.
11. During hydrolysis servo 1 with valve 1 was switched to the position B and syringe pump sucked in volume due to variable TRANSPORT.
12. Immediately after that control unit detected that the time period TIME_H is over, servo 1 with valve 1 was switched to the position A and the syringe pump pushed the sample out to the detection part.
13. Control unit gave an order for detection to the special unit which carried out the detection.

14. After the measurement the pump 3 pushed all the liquid out of the detection cell to the waste cell.

Rinsing Procedure

1. Servo 3 with valve 3 was switched to the position B, servo 1 with valve 1 was switched to the position B and syringe pump 1 sucked in wash solution with the maximal volume of the syringe pump.
2. Servo 1 with valve 1 was switched to the position A and syringe pump 1 pushed the rinse fluid out.
3. Servo 2 with valve 2 was switched to the position C and syringe pump 1 sucked the required volume back in for rinsing the hydrolysis cell.
4. Syringe pump 1 after 5 seconds pushed liquid out to the zero volume of syringe pump 1.
5. Servo 3 with valve 3 was switched to the position A, servo 1 with valve 1 was switched to the position B and syringe pump 1 sucked air in to the maximal volume of syringe pump 1.
6. Servo 1 with valve 1 was switched to the position A, syringe pump 1 pushed rinse fluid out to the detection cell. Pump 3 sucked out the wash liquid to waste.

© 2015 by the authors; licensee MDPI, Basel, Switzerland. This article is an open access article distributed under the terms and conditions of the Creative Commons Attribution license (http://creativecommons.org/licenses/by/4.0/).
